# Supplementary material for: Cross-cultural evaluation of the French version of the Delusion Assessment Scale (DAS) and Psychotic Depression Assessment Scale (PDAS)
Source: PLoS One. 2021 Apr 26;16(4):e0250492. doi: 10.1371/journal.pone.0250492 (PMC8075211; doi:10.1371/journal.pone.0250492)
Supplement: S1 Table — Spearman correlation coefficients between HDRS and BPRS items making up the PDAS scale. * Corrected item-total correlations. Significant correlations (p<0.05) are in bold. (DOCX) [file pone.0250492.s001.docx]

**S1 Table: Inter-item and** **corrected item-total correlations for PDAS**

|  | HDRS1 | HDRS2 | HDRS7 | HDRS8 | HDRS10 | HDRS13 | BPRS3 | BPRS11 | BPRS12 | BPRS15 | BPRS16 |
| --- | --- | --- | --- | --- | --- | --- | --- | --- | --- | --- | --- |
| HDRS1 | 1.00 |  |  |  |  |  |  |  |  |  |  |
| HDRS2 | **0.31** | 1.00 |  |  |  |  |  |  |  |  |  |
| HDRS7 | **0.26** | 0.12 | 1.00 |  |  |  |  |  |  |  |  |
| HDRS8 | **0.28** | **0.21** | **0.33** | 1.00 |  |  |  |  |  |  |  |
| HDRS10 | **0.35** | **0.22** | 0.18 | 0.17 | 1.00 |  |  |  |  |  |  |
| HDRS13 | **-0.22** | -0.17 | 0.16 | 0.04 | **0.22** | 1.00 |  |  |  |  |  |
| BPRS3 | 0.17 | 0.03 | **0.25** | **0.27** | -0.00 | -0.05 | 1.00 |  |  |  |  |
| BPRS11 | 0.11 | -0.13 | 0.03 | 0.07 | -0.04 | 0.04 | 0.13 | 1.00 |  |  |  |
| BPRS12 | 0.06 | -0.04 | 0.02 | 0.05 | 0.03 | -0.08 | 0.16 | -0.01 | 1.00 |  |  |
| BPRS15 | 0.01 | -0.06 | **0.22** | 0.11 | 0.18 | 0.15 | 0.16 | -0.00 | **0.29** | 1.00 |  |
| BPRS16 | -0.07 | -0.05 | 0.14 | 0.04 | **-0.21** | -0.11 | **0.68** | -0.08 | 0.03 | 0.17 | 1.00 |
| PDAS Total* | 0.28 | 0.12 | 0.43 | 0.39 | 0.25 | -0.02 | 0.43 | 0.02 | 0.10 | 0.28 | 0.13 |

Spearman correlation coefficients between HDRS and BPRS items making up the PDAS scale.

* Corrected item-total correlations

Significant correlations (p<0.05) are in bold.
