# Supplementary material for: Cross-cultural evaluation of the French version of the Delusion Assessment Scale (DAS) and Psychotic Depression Assessment Scale (PDAS)
Source: PLoS One. 2021 Apr 26;16(4):e0250492. doi: 10.1371/journal.pone.0250492 (PMC8075211; doi:10.1371/journal.pone.0250492)
Supplement: S2 Table — Spearman correlation coefficients between HDRS and BPRS items making up the PDAS scale. * Corrected item-total correlations. Significant correlations (p<0.05) are in bold. (DOCX) [file pone.0250492.s002.docx]

**S2 Table: Inter-item and** **corrected item-total correlations for DAS**

|  | DAS_i1 | DAS_i2 | DAS_i3 | DAS_i4 | DAS_i5 | DAS_i6 | DAS_i7 | DAS_i8 | DAS_i9 | DAS_i10 | DAS_i11 | DAS_i12 | DAS_i13 | DAS_i14 | DAS_i15 |
| --- | --- | --- | --- | --- | --- | --- | --- | --- | --- | --- | --- | --- | --- | --- | --- |
| DAS_i1 | 1.00 |  |  |  |  |  |  |  |  |  |  |  |  |  |  |
| DAS_i2 | **0.68** | 1.00 |  |  |  |  |  |  |  |  |  |  |  |  |  |
| DAS_i3 | **0.32** | **0.38** | 1.00 |  |  |  |  |  |  |  |  |  |  |  |  |
| DAS_i4 | 0.16 | 0.15 | 0.09 | 1.00 |  |  |  |  |  |  |  |  |  |  |  |
| DAS_i5 | **0.46** | **0.48** | **0.38** | **0.30** | 1.00 |  |  |  |  |  |  |  |  |  |  |
| DAS_i6 | **0.37** | **0.33** | **0.31** | 0.19 | **0.47** | 1.00 |  |  |  |  |  |  |  |  |  |
| DAS_i7 | **0.29** | **0.24** | **0.28** | 0.04 | **0.37** | **0.33** | 1.00 |  |  |  |  |  |  |  |  |
| DAS_i8 | **0.36** | **0.23** | **0.24** | 0.11 | **0.28** | 0.12 | 0.08 | 1.00 |  |  |  |  |  |  |  |
| DAS_i9 | **0.25** | **0.24** | **0.27** | -0.08 | **0.33** | 0.16 | -0.01 | **0.34** | 1.00 |  |  |  |  |  |  |
| DAS_i10 | **0.25** | **0.31** | **0.22** | 0.13 | **0.29** | **0.21** | 0.01 | 0.19 | **0.32** | 1.00 |  |  |  |  |  |
| DAS_i11 | **0.24** | **0.21** | **0.25** | 0.16 | **0.24** | **0.21** | 0.09 | 0.14 | 0.16 | **0.53** | 1.00 |  |  |  |  |
| DAS_i12 | 0.12 | **0.23** | 0.14 | 0.15 | 0.19 | 0.00 | -0.07 | **0.45** | 0.18 | **0.29** | **0.31** | 1.00 |  |  |  |
| DAS_i13 | **0.27** | **0.27** | 0.06 | **0.21** | 0.18 | 0.11 | -0.02 | **0.48** | 0.18 | **0.36** | **0.45** | **0.70** | 1.00 |  |  |
| DAS_i14 | 0.09 | 0.11 | 0.07 | 0.20 | 0.19 | 0.14 | -0.14 | **0.42** | 0.15 | 0.12 | 0.07 | **0.47** | **0.49** | 1.00 |  |
| DAS_i15 | -0.04 | 0.03 | 0.06 | 0.07 | **-0.22** | 0.03 | **-0.29** | 0.20 | -0.09 | 0.19 | 0.01 | **0.35** | **0.40** | **0.37** | 1.00 |
| DAS Total* | 0.54 | 0.54 | 0.43 | 0.25 | 0.56 | 0.40 | 0.14 | 0.53 | 0.35 | 0.49 | 0.42 | 0.51 | 0.58 | 0.40 | 0.15 |

Spearman correlation coefficients between HDRS and BPRS items making up the PDAS scale.

* Corrected item-total correlations

Significant correlations (p<0.05) are in bold.
