# Supplementary material for: Cross-cultural evaluation of the French version of the Delusion Assessment Scale (DAS) and Psychotic Depression Assessment Scale (PDAS)
Source: PLoS One. 2021 Apr 26;16(4):e0250492. doi: 10.1371/journal.pone.0250492 (PMC8075211; doi:10.1371/journal.pone.0250492)
Supplement: S3 Table — Spearman correlation coefficients between PDAS, DAS, HDRS, BPRS, CGI and SAPS scales. Significant correlations (p<0.05) are in bold. PDAS 1 = Depression; PDAS 2 = Emotional withdrawal/ Blunted affect; PDAS 3 = Fatigue/activities; PDAS 4 = Psychotic symptoms; PDAS 5 = Suspiciousness; DAS 1 = Delusional conviction; DAS 2 = Disorganization/ Mood congruence; DAS 3 = Bizarreness; DAS 4 = Acting irrationally during interview; DAS 5 = Places/situations involved; BPRS 1 = BPRS delusions hallucinations; BPRS 2 = BPRS hebephrenic; BPRS 3 = BPRS paranoia; BPRS 4 = BPRS melancholia anxious; BPRS 5 = BPRS acute psychotic; SAPS 1 = SAPS hallucinations; SAPS 2 = SAPS delusions; SAPS 3 = SAPS bizarre behavior; SAPS 4 = SAPS positive formal thought disorder. (DOCX) [file pone.0250492.s003.docx]

**S3 Table: Convergent validity for PDAS and DAS scales**

|  | PDAS total | PDAS 1 | PDAS 2 | PDAS 3 | PDAS 4 | PDAS 5 | DAS total | DAS 1 | DAS 2 | DAS 3 | DAS 4 | DAS 5 | HDRS total | BPRS total | BPRS 1 | BPRS 2 | BPRS 3 | BPRS 4 | BPRS 5 | CGI | SAPS total | SAPS 1 | SAPS 2 | SAPS 3 |
| --- | --- | --- | --- | --- | --- | --- | --- | --- | --- | --- | --- | --- | --- | --- | --- | --- | --- | --- | --- | --- | --- | --- | --- | --- |
| PDAS total | 1.00 |  |  |  |  |  |  |  |  |  |  |  |  |  |  |  |  |  |  |  |  |  |  |  |
| PDAS 1 | **0.57** | 1.00 |  |  |  |  |  |  |  |  |  |  |  |  |  |  |  |  |  |  |  |  |  |  |
| PDAS 2 | **0.47** | 0.01 | 1.00 |  |  |  |  |  |  |  |  |  |  |  |  |  |  |  |  |  |  |  |  |  |
| PDAS 3 | **0.45** | 0.05 | 0.02 | 1.00 |  |  |  |  |  |  |  |  |  |  |  |  |  |  |  |  |  |  |  |  |
| PDAS 4 | **0.56** | 0.04 | 0.17 | 0.10 | 1.00 |  |  |  |  |  |  |  |  |  |  |  |  |  |  |  |  |  |  |  |
| PDAS 5 | **0.31** | -0.01 | 0.02 | 0.05 | 0.01 | 1.00 |  |  |  |  |  |  |  |  |  |  |  |  |  |  |  |  |  |  |
| DAS total | **0.47** | **0.21** | 0.10 | 0.10 | **0.43** | **0.27** | 1.00 |  |  |  |  |  |  |  |  |  |  |  |  |  |  |  |  |  |
| DAS 1 | **0.33** | **0.40** | -0.01 | 0.03 | 0.18 | 0.10 | **0.75** | 1.00 |  |  |  |  |  |  |  |  |  |  |  |  |  |  |  |  |
| DAS 2 | **0.31** | -0.02 | 0.12 | 0.01 | **0.34** | **0.34** | **0.70** | **0.19** | 1.00 |  |  |  |  |  |  |  |  |  |  |  |  |  |  |  |
| DAS 3 | **0.39** | 0.12 | 0.06 | 0.15 | **0.45** | 0.15 | **0.65** | **0.34** | **0.38** | 1.00 |  |  |  |  |  |  |  |  |  |  |  |  |  |  |
| DAS 4 | **042** | 0.14 | **0.23** | 0.16 | 0.06 | **0.56** | **0.34** | **0.22** | 0.18 | 0.20 | 1.00 |  |  |  |  |  |  |  |  |  |  |  |  |  |
| DAS 5 | 0.04 | 0.00 | -0.01 | -0.00 | **0.23** | -0.18 | **0.49** | **0.30** | **0.23** | **0.26** | -0.08 | 1.00 |  |  |  |  |  |  |  |  |  |  |  |  |
| HDRS total | **0.58** | **0.54** | 0.07 | **0.48** | 0.13 | 0.08 | **0.29** | **0.34** | 0.01 | **0.24** | 0.13 | 0.08 | 1.00 |  |  |  |  |  |  |  |  |  |  |  |
| BPRS total | **0.82** | **0.39** | **0.51** | **0.25** | **0.49** | **0.34** | **0.50** | **0.34** | **0.31** | **0.42** | **0.46** | 0.11 | **0.42** | 1.00 |  |  |  |  |  |  |  |  |  |  |
| BPRS 1 | **0.66** | 0.02 | **0.28** | 0.11 | **0.83** | **0.47** | **0.53** | **0.25** | **0.47** | **0.47** | **0.38** | 0.11 | 0.15 | **0.71** | 1.00 |  |  |  |  |  |  |  |  |  |
| BPRS 2 | **0.64** | **0.27** | **0.79** | 0.10 | **0.27** | 0.14 | **0.35** | **0.25** | **0.25** | **0.21** | **0.38** | 0.10 | 0.22 | **0.78** | **0.45** | 1.00 |  |  |  |  |  |  |  |  |
| BPRS 3 | **0.35** | 0.02 | 0.12 | 0.10 | -0.01 | **0.89** | **0.28** | 0.08 | **0.32** | 0.19 | **0.65** | -0.19 | 0.11 | **0.45** | **0.43** | **0.28** | 1.00 |  |  |  |  |  |  |  |
| BPRS 4 | **0.35** | **0.59** | -0.08 | **0.28** | -0.09 | -0.00 | 0.13 | **0.27** | -0.12 | 0.03 | 0.10 | 0.10 | **0.52** | **0.43** | -0.05 | 0.08 | -0.01 | 1.00 |  |  |  |  |  |  |
| BPRS 5 | **0.25** | 0.16 | 0.11 | 0.15 | **0.23** | -0.13 | 0.19 | 0.16 | 0.10 | 0.15 | 0.07 | 0.04 | 0.15 | **0.51** | **0.24** | **0.28** | -0.01 | **0.32** | 1.00 |  |  |  |  |  |
| CGI | **0.34** | **0.39** | **0.23** | -0.04 | 0.15 | -0.04 | **0.48** | **0.48** | 0.19 | 0.16 | 0.13 | **0.41** | **0.46** | **0.32** | 0.11 | **0.34** | -0.00 | **0.30** | 0.05 | 1.00 |  |  |  |  |
| SAPS total | **0.23** | **-0.26** | **0.24** | 0.08 | **0.59** | 0.04 | **0.33** | 0.03 | **0.38** | **0.31** | 0.12 | 0.20 | -0.08 | **0.33** | **0.59** | **0.25** | 0.02 | -0.18 | **0.34** | 0.07 | 1.00 |  |  |  |
| SAPS 1 | **0.45** | 0.04 | 0.11 | 0.13 | **0.83** | -0.06 | **0.37** | 0.16 | **0.30** | **0.43** | -0.06 | 0.19 | 0.13 | **0.39** | **0.64** | **0.22** | -0.11 | -0.03 | **0.26** | 0.17 | **0.57** | 1.00 |  |  |
| SAPS 2 | 0.08 | 0.15 | -0.11 | 0.03 | -0.05 | 0.15 | **0.37** | **0.61** | 0.01 | 0.17 | 0.10 | 0.01 | **0.21** | 0.16 | 0.09 | 0.09 | 0.13 | 0.16 | 0.01 | 0.19 | -0.02 | -0.03 | 1.00 |  |
| SAPS 3 | 0.03 | -0.17 | 0.04 | 0.06 | 0.03 | **0.22** | 0.04 | -0.07 | 0.06 | 0.17 | 0.18 | -0.13 | 0.00 | 0.16 | 0.15 | 0.03 | **0.28** | -0.07 | **0.35** | -0.16 | **0.41** | 0.04 | -0.01 | 1.00 |
| SAPS 4 | -0.17 | **-0.32** | 0.03 | -0.05 | 0.12 | -0.12 | -0.02 | -0.10 | 0.01 | 0.02 | -0.00 | 0.09 | **-0.21** | -0.12 | 0.10 | -0.02 | -0.13 | **-0.28** | 0.08 | -0.06 | **0.51** | 0.06 | -0.16 | **0.24** |

Spearman correlation coefficients between PDAS, DAS, HDRS, BPRS, CGI and SAPS scales.

Significant correlations (p<0.05) are in bold.

PDAS 1= Depression ; PDAS 2= Emotional withdrawal/ Blunted affect ; PDAS 3= Fatigue/activities ; PDAS 4= Psychotic symptoms ; PDAS 5= Suspiciousness ; DAS 1= Delusional conviction ; DAS 2= Disorganization/ Mood congruence ; DAS 3= Bizarreness ; DAS 4= Acting irrationally during interview ; DAS 5= Places/situations involved ; BPRS 1=BPRS delusion hallucination ; BPRS 2= BPRS hebephrenic; BPRS 3=BPRS paranoia; BPRS 4=BPRS melancholia anxious; BPRS 5=BPRS acute psychotic; SAPS 1=SAPS hallucination; SAPS 2=SAPS delusion; SAPS 3=SAPS bizarre behavior; SAPS 4=SAPS positive formal thought disorder.
