# Supplementary material for: Cross-cultural evaluation of the French version of the Delusion Assessment Scale (DAS) and Psychotic Depression Assessment Scale (PDAS)
Source: PLoS One. 2021 Apr 26;16(4):e0250492. doi: 10.1371/journal.pone.0250492 (PMC8075211; doi:10.1371/journal.pone.0250492)
Supplement: S1 File — (DOC) [file pone.0250492.s004.doc]

**ECHELLE D’EVALUATION DU DELIRE (DAS)**

**I. CONVICTION délirante**

*Les items relatifs à la conviction délirante évaluent le délire le plus prononcé.*

*Force de la croyance et capacité à douter de celle-ci ; influence de la croyance sur le comportement. Sont inclues les informations obtenues grâce à l’entretien avec le patient et les constatations faites par des tiers en dehors de l’entretien.*

**Item 1. Sentiment subjectif de certitude** *(évaluation basée sur les éléments exprimés par le patient durant* *l’entretien)*

**1** Capacité à exprimer un doute au sujet de la croyance (p. ex., reconnaissance du fait que la croyance pourrait résulter d’une maladie) malgré le refus d’accepter entièrement la croyance comme inexacte.

**2** Quasi-certitude que la croyance est vraie ; tous les doutes ne sont que transitoires;

**3** Conviction absolue et inébranlable.

**Item 2. Distance critique** *(cotation par l’évaluateur des réponses lors de la confrontation à la réalité, par opposition avec l’item I qui évalue le niveau subjectif de conviction).*

**1** La confrontation conduit à une modification de la croyance

**2** La confrontation conduit à considérer d’autres explications ou à modifier certains aspects de la croyance, mais la croyance centrale est inchangée

**3** La confrontation n’a aucun effet sur la croyance et ne conduit pas à considérer d’autres explications.

**Item 3. Actions basées sur la croyance** *(coté à partir de l’anamnèse et l’explication du comportement antérieur du patient au cotateur; évalue aussi si le jugement est affecté par la croyance)*

**1** La croyance n’influence pas les actions

**2** Agit ou a agi en fonction de la croyance, mais ces actions n’ont pas de conséquences significatives telles que détériorer le fonctionnement et/ou affecter les autres (p. ex., avoir un comportement méfiant à l’égard de l’équipe hospitalière ; changer les serrures des portes, consultations itératives de médecins en raison de plaintes somatiques, se plaindre des voisins à la police).

**3** Les actions démontrent une altération manifeste du jugement ; les actions ont un impact significatif sur la vie du patient avec des conséquences négatives pour le patient ou les autres (p. ex., actions qui ont conduit directement à une hospitalisation, telles que refuser de manger ou faire une tentative de suicide).

**Item 5. Pression temporelle** *(coté en utilisant les observations, y compris celles des autres)*

**1** La plupart de la journée est passée sans être concerné par la croyance

**2** Suffisamment préoccupé par la croyance dans la journée pour interférer avec la réalisation des activités comme les tâches quotidiennes courantes ou le travail

**3** La préoccupation liée à la croyance est quasi constante et interfère avec la réalisation de toutes ou presque toutes les responsabilités simples.

**Item 6. Pression temporelle durant l’entretien** *(coté à partir de l’entretien)*

**1** Les pensées sur la croyance ne font pas irruption durant l’entretien et engager une discussion étendue à d’autres sujets est possible.

**2** La croyance ou les idées reliées font irruption durant l’entretien, mais la diversion vers d’autres sujets est possible.

**3** La croyance ou les pensées reliées interrompent de façon répétée l’entretien et l’orientation de la discussion sur d’autres sujets au-delà de quelques instants ou phrases n’est pas réalisable.

**Item 7. Pression émotionnelle** *(coté principalement d’après l’entretien mais peut intégrer les observations des autres)*

**1** La croyance n’engendre pas de détresse émotionnelle ou d’inquiétudes évidentes

**2** La discussion sur la croyance engendre une détresse émotionnelle subjective ou objective (par exemple anxiété ou dépression)

**3** La discussion sur la croyance cause une détresse marquée conduisant à des expressions intenses de l’affect (p. ex., crainte prononcée, pleurs ou peur manifestes)

**II. Désorganisation* / congruenCe***

**La désorganisation prend toujours en compte plusieurs idées délirantes, si elles sont présentes.*

**Ce construit n’évalue pas l’organisation des processus de pensée en général.*

**Item 8. Personnes et objets impliqués**

**1** Le nombre d’objets impliqués est limité (p. ex., organe(s) malade(s) dans le cas de délires somatiques, organismes et leurs employés, p. ex., services secrets, voisins d’à côté, une équipe médicale spécifique ou un téléphone spécifique mis sur écoute)

**2** Le nombre de personne ou d’organismes impliqués ne peut être défini clairement mais apparaît limité par des caractéristiques identifiables et peut être organisé autour d’un thème central (p. ex., croire que les collègues de travail conspirent contre l’individu et peuvent en avoir enrôlé d’autres)

**3** L’évaluateur ne peut obtenir une description claire et complète du délire ; il n’y a pas de caractéristiques définies qui limitent le nombre de personne ou d’organismes impliqués dans la croyance. Pour les délires somatiques les organes ou les maladies sur lesquels se focalisent les plaintes ne peuvent être identifiés.

**Item 12. Cohérence interne** *(cotations basées sur l’entretien)*

**1** Les croyances uniques ou multiples sont clairement reliées à une idée délirante principale ou à un thème unique (p.ex., sentiments culpabilité, de dévalorisation et de mériter d’être puni pour un écart de conduite sexuelle dans un passé lointain).

**2** De multiples idées délirantes sont présentes reliées à un thème central. Toutefois, les idées impliquent plus d’une fausse supposition et ne sont pas clairement reliées (p.ex., le patient sera puni par le service des impôts pour avoir bénéficié d’une exemption d’impôt supplémentaire il y a 20 ans et les voisins espionnent et contrôlent son comportement pour la police).

**3** De multiples idées délirantes qui ne peuvent pas être connectées à un seul thème (p.ex., délire d’être persécuté sans raison apparente, que ses organes génitaux aient été retirés et de recevoir des messages par télépathie)

**Item 13. Intégration cognitive** *(cotée d’après l’entretien)*

**1** Le patient peut expliquer comment de multiples idées délirantes sont reliées à une croyance unique principale ou décrit un délire unique qui constitue une histoire cohérente

**2** Il existe de multiples parties d’une idée délirante unique, ou de multiples idées délirantes, que le patient essaie d’intégrer mais il ne peut pas expliquer de manière cohérente comment les multiples idées sont reliées.

**3** Le patient reconnaît le manque de lien entre les multiples idées délirantes avec un thème unique ou une croyance principale, et ne peut pas fournir ou ne fournira pas d’explication.

**Item 14. Continuité temporelle de(s) l’idée(s) délirante(s) la plus prononcée(s)** *(cotée en utilisant l ‘anamnèse du patient et l’évaluation durant l’entretien)*

**1** La (les) même(s) idée(s) délirante(s) a/ont été maintenue (s) régulièrement durant un épisode morbide sans ajout d’idées nouvelles

**2** Durant l’épisode actuel ou l’entretien, il y a eu une idée délirante principale régulièrement présente, mais avec apparition de quelques modifications ou émergence d’un autre délire. Des idées additionnelles transitoires auraient pu se développer ou se résoudre.

**3** Le contenu du délire principal a changé significativement durant l’épisode morbide ou a été remplacé par un délire différent (p.ex., la peur d’être suivi dans le quartier est devenue une peur d’être empoisonné par un autre patient).

**Item 15. Congruence de l’humeur** *(Evaluer seulement le délire le* plus *prononcé. Evaluer à quel degré la croyance délirante est congruente avec la perturbation de l’humeur)* **.*

**1** Délires de culpabilité, désespoir ou nihilisme ; délires somatiques (par convention) ; délire paranoïaque dans lequel la persécution est attribuée à un méfait personnel réel ou imaginé ; croyance que la dépression actuelle n’est pas curable et que le patient aura besoin d’une hospitalisation sa vie durant).

**2** La croyance délirante est cohérente avec le thème du trouble de l’humeur à travers une défaillance personnelle du patient, mais le lien est ténu (délire paranoïaque à propos d’une conspiration pour résilier son contrat de travail parce que la dépression a amoindri ses capacités, délire paranoïaque que ses enfants volent de l’argent parce que le patient a été indisponible pour ses petits-enfants).

**3** Il n’y a aucun lien entre la croyance délirante et le trouble de l’humeur (p.ex., un délire paranoïaque dans lequel le patient croit que la persécution est injuste et n’est pas due à un méfait personnel ou à un attribut négatif).

***** *La cotation ne prend pas en compte si l’idée délirante ou le symptôme thymique est plus prononcé ; p.ex., les idées délirantes peuvent être plus ou moins prononcées que les symptômes thymiques, quelle que soit la congruence des délires.*

**III. BIZARRERIE**

*Les items bizarrerie prennent en compte tous les délires, avec un focus sur le plus invraisemblable.*

**Item 10. Distorsion avec la réalité consensuelle** **(caractère invraisemblable)**

**1** Peut être considéré comme un jugement de valeur extrême ou éloigné du possible mais n’est pas manifestement absurde du fait d’une impossibilité physique (p.ex., délire somatique d’avoir un cancer ou délire paranoïaque d’être suivi)

**2** Le contenu de la croyance est manifestement invraisemblable mais n’est pas physiquement impossible (p.ex., délire somatique d’avoir été empoisonné par un persécuteur, d’être victime d’un complot de ses collègues de travail visant à un licenciement, ou délire paranoïaque qu’un voisin a mis le téléphone sur écoute ou a piraté l’ordinateur du patient)

**3** La croyance est manifestement absurde et physiquement impossible (p.ex., délire que les pensées sont subtilisées ou implantées électroniquement, d’avoir une micropuce implantée dans le cerveau)

**Item 11. Relation avec le contexte culturel**

**1** La croyance est compatible avec le contexte culturel du patient et pourrait sembler plausible à des personnes de la même culture (p.ex., conviction d’être persécuté en raison de sa race ou de son genre)

**2** Bien que des membres issus de la même culture ne puissent considérer la croyance comme plausible, la croyance est directement reliée au contexte culturel (p.ex., le patient croit avoir été désigné pour être persécuté pour donner un exemple à l’ensemble des personnes de son ethnie/sa religion ou parce qu’il a été choisi pour une mission spéciale par Dieu)

**3** La croyance est complètement déviante par rapport à celle adoptée par les autres membres du groupe culturel du patient (p.ex., croyance que l’individu a été choisi pour coloniser l’espace pour sa race).

**IV. Actions irrationnelles envers l’évaluateur**

**Item 4. Actions irrationnelles envers l’évaluateur** *(coté à partir des observations de l’évaluateur, incluant les réponses aux tentatives de clarification et à la confrontation)*

**1** N’est pas méfiant vis-à-vis de l’évaluateur

**2** Méfiant comme mettant en doute les motifs de l’évaluateur (p. ex., refus de fournir des informations personnelles au clinicien ou demande de quitter l’hôpital sans traitement compte tenu de l’absence de maladie)

**3** Refus de répondre aux questions personnelles même les plus simples ou mutique.

**V. Lieux/Situations**

**Item 9. Lieux/Situations dans lesquels l’idée délirante est la plus prononcée**

**1** L’idée délirante est limitée à un contexte/lieu unique (p. ex., travail/maison/voyage en métro). La paranoïa est limitée à la maison ou au travail mais n’implique pas l’hôpital. Parce que le patient se déplace physiquement en différents endroits, un délire somatique circonscrit est coté 1.

**2** Les idées délirantes surviennent dans des contextes ou des lieux multiples qui ont des caractéristiques communes identifiables (p.ex., le voisinage, l’hôpital et d’autres lieux compatibles avec un thème délirant spécifique tel que la persécution basée sur l’identité ethnique)

**3** Il n’y a pas de caractéristiques définies qui limitent les lieux/situations qui peuvent être impliquées dans les croyances.

Modified from Meyers BS et al. A delusion assessment scale for psychotic major depression: Reliability, validity, and utility. Biol Psychiatry. déc 2006;60(12):1336‑42 under a CC BY license, printed with permission from Pr. Barnett S. Meyers.
