# Supplementary material for: Cross-cultural evaluation of the French version of the Delusion Assessment Scale (DAS) and Psychotic Depression Assessment Scale (PDAS)
Source: PLoS One. 2021 Apr 26;16(4):e0250492. doi: 10.1371/journal.pone.0250492 (PMC8075211; doi:10.1371/journal.pone.0250492)
Supplement: Attachment — Submitted filename: Response to Reviewers.docx [file pone.0250492.s005.docx]

**Response to the academic editor**

*1.) Please ensure that your manuscript meets PLOS ONE's style requirements, including those for file naming. The PLOS ONE style templates can be found at*

[*https://journals.plos.org/plosone/s/file?id=wjVg/PLOSOne_formatting_sample_main_body.pdf*](https://journals.plos.org/plosone/s/file?id=wjVg/PLOSOne_formatting_sample_main_body.pdf) *and*

[*https://journals.plos.org/plosone/s/file?id=ba62/PLOSOne_formatting_sample_title_authors_affiliations.pdf*](https://journals.plos.org/plosone/s/file?id=ba62/PLOSOne_formatting_sample_title_authors_affiliations.pdf)

 Style requirement has been checked and changed when required.

*2.) Please describe in your methods section how capacity to consent was determined for the participants in this study.*

This part of the methods section has been developed as follow:

“A psychiatrist explained the aims and procedures to the patients and collected their consent to participate in the study. All patients received written information about the study. They could refuse the use of the data collected at any time.”

*3.) One of the noted authors is a group or consortium: French DAS/PDAS group.*

*In addition to naming the author group, please list the individual authors and affiliations within this group in the acknowledgments section of your manuscript.*

*Please also indicate clearly a lead author for this group along with a contact email address.*

Membership of the French DAS/PDAS group is provided in the acknowledgments and the lead leader for this group is clearly indicated:

“The authors thank Barnett S. Meyers for allowing them to validate the French version of the DAS scale and the membership of the French DAS/PDAS group: A. Amour, E. Arango, F. Blanchard, L. Morel and N. Zouaoui (Centre Hospitalier Spécialisé Sainte-Marie Le Puy-en-Velay), D. Dhenain, I. Gaume and S.A. Nechifor (Centre Hospitalier Spécialisé Sainte-Marie Clermont-Ferrand), J. Chabert, I. Jalenques (leader of the group, [ijalenques@chu-clermontferrand.fr](mailto:ijalenques@chu-clermontferrand.fr)), S. Lauron, C. Rachez, F. Rondepierre and P.A. Thevenet (Centre Hospitalier Universitaire Clermont-Ferrand, Service de Psychiatrie de l’Adulte et Psychologie Médicale), B. Pereira (Centre Hospitalier Universitaire Clermont-Ferrand, Direction de la Recherche Clinique et de l’Innovation), U. Tauveron Jalenques and Jeffrey Watts (Université Clermont Auvergne).”

**Response to reviewers**

***Comments to the Author*** *1. Is the manuscript technically sound, and do the data support the conclusions?

The manuscript must describe a technically sound piece of scientific research with data that supports the conclusions. Experiments must have been conducted rigorously, with appropriate controls, replication, and sample sizes. The conclusions must be drawn appropriately based on the data presented.*

*Reviewer #1: Yes*

*Reviewer #2: Yes*

*2. Has the statistical analysis been performed appropriately and rigorously?*

*Reviewer #1: Yes*

*Reviewer #2: Yes*

*3. Have the authors made all data underlying the findings in their manuscript fully available?

The* [*PLOS Data policy*](http://www.plosone.org/static/policies.action#sharing) *requires authors to make all data underlying the findings described in their manuscript fully available without restriction, with rare exception (please refer to the Data Availability Statement in the manuscript PDF file). The data should be provided as part of the manuscript or its supporting information, or deposited to a public repository. For example, in addition to summary statistics, the data points behind means, medians and variance measures should be available. If there are restrictions on publicly sharing data—e.g. participant privacy or use of data from a third party—those must be specified.*

*Reviewer #1: Yes*

*Reviewer #2: Yes*

*4. Is the manuscript presented in an intelligible fashion and written in standard English?

PLOS ONE does not copyedit accepted manuscripts, so the language in submitted articles must be clear, correct, and unambiguous. Any typographical or grammatical errors should be corrected at revision, so please note any specific errors here.*

*Reviewer #1: Yes*

*Reviewer #2: Yes*

*5. Review Comments to the Author

Please use the space provided to explain your answers to the questions above. You may also include additional comments for the author, including concerns about dual publication, research ethics, or publication ethics. (Please upload your review as an attachment if it exceeds 20,000 characters)*

*Reviewer #1: Major Depression with psychotic features (psychotic depression) is a serious illness for which the diagnosis is often missed. Two important rating scales have been developed to help clinicians diagnose this condition. The authors present data from a study to validate the French version of these two important scales that can aid clinicians in making an accurate diagosis -the PDAS and the DAS. The manuscript is well written and the data clearly presented. I had a few suggestions and questions which should be addressed:*

*1. page 6- "The PDAS Instrument" should be in BOLD.*
“The PDAS Instrument” has been bolded page 6.

*2. page 9 - The frequency of hallucinations was rather high (45%) for patients with psychotic depression. Most studies report lower rates (around 5% I think). Furthermore, I note that 90% of the patients had delusions, not 100%). The DAS and the PDAS were developed from the Study of the Pharmacotherapy of Psychotic Depression or STOP-PD. The investigators in that study required that the patients have a delusion. I also think their rate of hallucinations was much lower than your sample.*

*The concern I have is that those patients who present with hallucinations without delusions may not have psychotic depression, but perhaps a different disorder, e.g. PTSD. I think you should point out the differences between your sample and the STOP-PD sample from which the 2 scales are based. Ideally, I would prefer to see an analysis on only patients who had a delusion (as in STOP-PD).*

We thank you for this remark which highlighted an important error when writing our article: as in STOP-PD, all patients had at least one clearly defined delusion and a score of ≥3 on the delusion severity rating item of the SADS (Schedule for Affective Disorders and Schizophrenia). This was an inclusion criteria in our study. Thus no patients had hallucinations without delusions and we confirmed that all patients had a diagnosis of major depressive disorder with psychotic features on the basis of DSM 5 criteria. We used the SAPS to classify the type of delusions and hallucinations but not to rate their severity. And the 90% patients with delusions and 45% with hallucinations were based on SAPS. We have removed theses muddled data from the article. However, we agreed that the proportion of our patients with hallucinations was superior to those observed in STOP-PD. Indeed, it could be due to difference in the population. Our BPRS, DAS and PDAS scores were similar to those in STOP-PD (Meyers et al. 2006) and to other article using PDAS (Köse Çinar et Østergaard 2018; Vermeulen et al. 2018). However, our patients had lower HDRS score (25.5 vs 29.8), had more depressive episodes (23% with a first episode in our study vs 30.1%), had more suicide attempts (32% vs 18.5) than patients in these studies. They are all inpatients (69% in STOP-PD, Meyers et al. 2009). This could be related to the higher proportion of our patients with hallucinations, as in a study of Gournellis (Gournellis et al. 2001) in which 36% of inpatients with psychotic depression had hallucinations.

As recommended, we pointed out these difference in the discussion.

*3. page 24- I believe you want to thank Barnett S. Meyers, M.D.*

We have corrected it.

*Gournellis, R., L. Lykouras, A. Fortos, P. Oulis, V. Roumbos, et G. N. Christodoulou. 2001. « Psychotic (Delusional) Major Depression in Late Life: A Clinical Study ». International Journal of Geriatric Psychiatry 16 (11): 1085‑91. https://doi.org/10.1002/gps.483.*

*Köse Çinar, Rugül, et Søren Dinesen Østergaard. 2018. « Validation of the Semi-Structured Psychotic Depression Assessment Scale (PDAS) Interview ». Acta Neuropsychiatrica 30 (3): 175‑80. https://doi.org/10.1017/neu.2017.15.*

*Meyers, Barnett S., Judith English, Michelle Gabriele, Catherine Peasley-Miklus, Moonseong Heo, Alastair J. Flint, Benoit H. Mulsant, Anthony J. Rothschild, et STOP-PD Study Group. 2006. « A Delusion Assessment Scale for Psychotic Major Depression: Reliability, Validity, and Utility ». Biological Psychiatry 60 (12): 1336‑42. https://doi.org/10.1016/j.biopsych.2006.05.033.*

*Meyers, Barnett S., Alastair J. Flint, Anthony J. Rothschild, Benoit H. Mulsant, Ellen M. Whyte, Catherine Peasley-Miklus, Eros Papademetriou, Andrew C. Leon, Moonseong Heo, et STOP-PD Group. 2009. « A Double-Blind Randomized Controlled Trial of Olanzapine plus Sertraline vs Olanzapine plus Placebo for Psychotic Depression: The Study of Pharmacotherapy of Psychotic Depression (STOP-PD) ». Archives of General Psychiatry 66 (8): 838‑47. https://doi.org/10.1001/archgenpsychiatry.2009.79.*

*Vermeulen, Tom, Lieve Lemey, Linda Van Diermen, Didier Schrijvers, Yamina Madani, Bernard Sabbe, Maarten J. A. Van Den Bossche, Roos C. van der Mast, et Søren D. Østergaard. 2018. « Clinical Validation of the Psychotic Depression Assessment Scale (PDAS) against Independent Global Severity Ratings in Older Adults ». Acta Neuropsychiatrica 30 (4): 203‑8. https://doi.org/10.1017/neu.2018.2.*

*Reviewer #2: This validation of the French version of the DAS and PDAS is a valuable addition to the literature. There are however some methodological issues that must be solved prior to publication. I have the following suggestions for improvement:

1. Abstract/Introduction: Both the abstract and the introduction focuses on the underdiagnosis/lack of recognition of psychotic depression. While this is certainly a clinical problem, neither the DAS nor the PDAS were developed to aid diagnosis of psychotic depression, but rather to MEASURE delusions and the overall severity of psychotic depression. Hence, the underdiagnosis angle is somewhat misplaced. This reviewer suggests increased emphasis on measurement instead. This is also more in line with the aim of the study: “The present study aimed to validate French versions of the DAS and PDAS after evaluation of their psychometric properties for use in measuring the severity of psychotic depression in MDDPsy patients.”*
Modifications have been done as recommended in the abstract and in the introduction sections (pages 3 and 4).

*2. Methods: “The psychiatrist completed the questionnaires after a routine interview
with patients shortly after hospital admission.” Which questionnaires? The DAS and PDAS are not questionnaires.*
We agreed with you remark and replaced “questionnaires” by “the DAS and PDAS scales”.

*3. Methods: Some of the exclusion criteria are quite strict, e.g. “meeting criteria for obsessive-compulsive disorder” and “history of drug or alcohol abuse in the past three months”. This has reduced the generalizability of the results and should be mentioned under the limitations. Relatedly, “taking a medication with known psychiatric effects” needs to be rephrased and explained.*
The exclusion criteria, as the inclusion criteria, were the same that those used in the STOP-PD study (Meyers et al. 2006; 2009). We agreed that this reduced the generalizability of the results and we mentioned it in the limitations. The terms “taking a medication with known psychiatric effects” were from the article of Meyers *et al*. (2006) which presented the DAS scale. This term has been changed “taking a medication which may induce psychiatric symptoms or disorders”.

*4. Methods: The font size of the subtitle “The PDAS instrument” should be increased.*
“The PDAS Instrument” has been bolded in the methods.

*5. Methods: “In addition, there exist validated French versions of the scales [25,26].” Actuelly, there is a publicly available French version of the PDAS available:* [*https://psychoticdepressionassessmentscale.com/french-pdas/*](https://psychoticdepressionassessmentscale.com/french-pdas/)

*Have the authors used this version inclusing the semi-structured interview? If not, it should be specified. This interview has been used in the two most recent validations of the PDAS:*
[*https://pubmed.ncbi.nlm.nih.gov/28535843/*](https://pubmed.ncbi.nlm.nih.gov/28535843/)
[*https://pubmed.ncbi.nlm.nih.gov/29501075/*](https://pubmed.ncbi.nlm.nih.gov/29501075/)
When the study started, the French version of the semi-structured interview for the PDAS was not available. For the PDAS, we used the complete HDRS and BPRS scales (and interviews) from which specific items of the PDAS were extracted. We added this specification in the assessment section of the method.

*6. Methods: How was the “General somatic” item of the PDAS rated? In most studies of the PDAS, a rescaling of this item to a 0-4 range was made. This reviewer suggests that the same is done for the present study. Similarly, the BPRS should be rescaled to 0-4 in all analyses to allow for comparison with other studies. See e.g. Østergaard SD, Meyers BS, Flint AJ, Mulsant BH, Whyte EM, Ulbricht CM, et al. Measuring psychotic depression. Acta Psychiatr Scand. mars 2014;129(3):211‑20.*

We thank you for this relevant remark. BPRS scores had not been rescaled; we did it and we have presented the update results in this new version. The descriptive statistics and score distributions of the PDAS (in the table 4) are the most impacted results.

*7. Methods: The authors place substantial emphasis on inter-item correlations, item-total correlations and inter-scale correlations. This reviewer is not convinced by the value of these analyses (such overlap can be a product of redundancy = items overlapping in content = the same psychopathology measured several times). Rather, the authors should focus on whether the individual items add unique information to the scale. This aspect (unidimensionality/scalability) is tested by item response theory analysis and the PDAS performs very well in this regard:*
[*https://pubmed.ncbi.nlm.nih.gov/23799875/*](https://pubmed.ncbi.nlm.nih.gov/23799875/)
[*https://pubmed.ncbi.nlm.nih.gov/28535843/*](https://pubmed.ncbi.nlm.nih.gov/28535843/)
[*https://pubmed.ncbi.nlm.nih.gov/29501075/*](https://pubmed.ncbi.nlm.nih.gov/29501075/)
[*https://pubmed.ncbi.nlm.nih.gov/25462426/*](https://pubmed.ncbi.nlm.nih.gov/25462426/) *I therefore strongly suggest that the authors add item response theory analyses and decrease the emphasis on the inter-item correlations, item-total correlations and inter-scale correlations – as they are not particularly meaningful/informative from a psychometric perspective.*

We thank the reviewer for the helpful and relevant comment. We agree that other approaches like that item response theory can be useful and informative for the analysis of the construct validity, especially for PDAS. According to the reviewer’s comment and to the COSMIN recommendations, this statistical analysis was conducted. The Loevinger coefficient has been estimated. As described in the revised manuscript, Loevinger coefficients of homogeneity derived from the Mokken analyses are 0.39 and 0.28 for DAS and PDAS scales, respectively. The predefined threshold for scalability (≥0.40) was not achieved although significant results (both < 0.001).

*8. Results: “Almost half of the patients (45%) had hallucinations and almost all (90%) had
delusions.” Why do not all patients have a delusion – as dictated by the inclusion criteria:
“Inclusion criteria were diagnosis of major depressive disorder with psychotic features on the basis of DSM 5 criteria [1], a delusional idea as defined in DSM-5 with a rating ≥3 (delusion definitely present) on the delusional rating item of the Schedule for Affective Disorders and Schizophrenia[20].”*

We thank you for this remark which highlighted an important error when writing our article: as in STOP-PD, all patients had at least one clearly defined delusion and a score of ≥3 on the delusion severity rating item of the SADS (Schedule for Affective Disorders and Schizophrenia). This was an inclusion criteria. We used the SAPS to classify the type of delusions and hallucinations but not to rate their severity. The 90% patients with delusions and 45% with hallucinations were based on SAPS. We have removed theses muddled data from the article.

*9. Discussion: Please be consistent in the use of decimal points: “With regard to the PDAS, the six items from the HDRS were divided into two dimensions, 1 and 3, which accounted, respectively, for 18.1 et 13% of variance; the five items from the BPRS were divided into three dimensions, 2, 4 and 5, which accounted, respectively, for 17.3, 11.8 et 10% of variance.”*
Modifications have been done page 20:

“*With regard to the PDAS, the six items from the HDRS were divided into two dimensions, 1 and 3, which accounted, respectively, for 18.1 et 13.0% of variance; the five items from the BPRS were divided into three dimensions, 2, 4 and 5, which accounted, respectively, for 17.3, 11.8 et 10.0% of variance.*”

*10. Discussion: This is related to comment no. 7: “The absence of inter-dimension correlations in the PDAS shows that the dimensions are independent of one another. This could be related to the fact that factors 1 and 3 are composed of items from the HDRS and the other three factors of items from the BPRS. This method of construction of the PDAS could also explain the moderate corrected item-total and the weak inter-item correlations.” Yes – but this reviewer would argue that the lacking correlation is actually a significant strentgh of the PDAS. We do not want to measure the same psychopathology several times = redundancy = the total score of a scale is not meaningful. Please revise accordingly.*

We thank the reviewer for the interesting comment. We perfectly agree. Interestingly, the complementary statistical analyses based on item response theory suggested by the reviewer confirmer the remark that the lacking correlation can be seen as a significant strength of the PDAS. The discussion have been revised as recommended.

*11. Discussion: “Further studies on diagnostic performance and responsiveness to change are needed before the French versions of the DAS and PDAS can be fully recommended to help guide the diagnosis or assess the efficacy of a treatment.” Again, The DAS and PDAS are not intended to be diagnostic tools. Furthermore, the responsiveness of the PDAS seems to be quite good:*
[*https://pubmed.ncbi.nlm.nih.gov/24439830/*](https://pubmed.ncbi.nlm.nih.gov/24439830/)
[*https://pubmed.ncbi.nlm.nih.gov/29501075/*](https://pubmed.ncbi.nlm.nih.gov/29501075/)
[*https://pubmed.ncbi.nlm.nih.gov/28535843/*](https://pubmed.ncbi.nlm.nih.gov/28535843/)
[*https://pubmed.ncbi.nlm.nih.gov/25462426/*](https://pubmed.ncbi.nlm.nih.gov/25462426/)
[*https://pubmed.ncbi.nlm.nih.gov/26496016/*](https://pubmed.ncbi.nlm.nih.gov/26496016/)

The limitation section of the abstract and the discussion page 24 have been modified as recommended

**“Limitations:** Interjudge reliability was calculated from a relatively small number of volunteers.”

“The present study has certain limitations. Interjudge reliability was calculated from the assessments of 26 patients who had consented to a second interview. This relatively small number of volunteers can be explained by the symptoms of MDDPsy. ~~In addition we did not perform studies of responsiveness to change or of~~ ~~diagnostic performance.~~”

The conclusion have also been modified as recommended:

“In conclusion, our study provides evidence of the good psychometric properties of the French versions of the DAS and PDAS. However, other studies are warranted to confirm the scalability of the PDAS and the generalizability of the results to more extensive population. This should help clinicians and researchers to assess cases of MDDPsy, which is a severe disorder, whose prognosis is marked by the risk of suicide and the lesser likelihood of patients achieving treatment response. The cross-cultural adaptation of this specific instrument should enable clinicians as of now to better assess the symptoms of MDDpsy, particularly psychotic features. It should also enable investigators to propose French-speaking persons with MDDPsy as participants in international collaboration research projects. In both cases, the response to treatment and prognosis of MDDPsy should be improved.”

*Meyers, Barnett S., Judith English, Michelle Gabriele, Catherine Peasley-Miklus, Moonseong Heo, Alastair J. Flint, Benoit H. Mulsant, Anthony J. Rothschild, et STOP-PD Study Group. 2006. « A Delusion Assessment Scale for Psychotic Major Depression: Reliability, Validity, and Utility ». Biological Psychiatry 60 (12): 1336‑42. https://doi.org/10.1016/j.biopsych.2006.05.033.*

*Meyers, Barnett S., Alastair J. Flint, Anthony J. Rothschild, Benoit H. Mulsant, Ellen M. Whyte, Catherine Peasley-Miklus, Eros Papademetriou, Andrew C. Leon, Moonseong Heo, et STOP-PD Group. 2009. « A Double-Blind Randomized Controlled Trial of Olanzapine plus Sertraline vs Olanzapine plus Placebo for Psychotic Depression: The Study of Pharmacotherapy of Psychotic Depression (STOP-PD) ». Archives of General Psychiatry 66 (8): 838‑47. https://doi.org/10.1001/archgenpsychiatry.2009.79.*

6. PLOS authors have the option to publish the peer review history of their article ([what does this mean?](https://journals.plos.org/plosone/s/editorial-and-peer-review-process#loc-peer-review-history)). If published, this will include your full peer review and any attached files.


If you choose “no”, your identity will remain anonymous but your review may still be made public.

**Do you want your identity to be public for this peer review?** For information about this choice, including consent withdrawal, please see our [Privacy Policy](https://www.plos.org/privacy-policy).

Reviewer #1: No

Reviewer #2: No

[NOTE: If reviewer comments were submitted as an attachment file, they will be attached to this email and accessible via the submission site. Please log into your account, locate the manuscript record, and check for the action link "View Attachments". If this link does not appear, there are no attachment files.]

While revising your submission, please upload your figure files to the Preflight Analysis and Conversion Engine (PACE) digital diagnostic tool, <https://pacev2.apexcovantage.com/>. PACE helps ensure that figures meet PLOS requirements. To use PACE, you must first register as a user. Registration is free. Then, login and navigate to the UPLOAD tab, where you will find detailed instructions on how to use the tool. If you encounter any issues or have any questions when using PACE, please email PLOS at [figures@plos.org](mailto:figures@plos.org). Please note that Supporting Information files do not need this step.

*In compliance with data protection regulations, you may request that we remove your personal registration details at any time.* [*(Remove my information/details)*](https://www.editorialmanager.com/pone/login.asp?a=r)*. Please contact the publication office if you have any questions.*
