# Supplementary material for: Cross-cultural evaluation of the French version of the Delusion Assessment Scale (DAS) and Psychotic Depression Assessment Scale (PDAS)
Source: PLoS One. 2021 Apr 26;16(4):e0250492. doi: 10.1371/journal.pone.0250492 (PMC8075211; doi:10.1371/journal.pone.0250492)
Supplement: Attachment — Submitted filename: Responses to reviewers.docx [file pone.0250492.s006.docx]

**Response to the academic editor**

*Journal Requirements:*

*Please review your reference list to ensure that it is complete and correct. If you have cited papers that have been retracted, please include the rationale for doing so in the manuscript text, or remove these references and replace them with relevant current references. Any changes to the reference list should be mentioned in the rebuttal letter that accompanies your revised manuscript. If you need to cite a retracted article, indicate the article’s retracted status in the References list and also include a citation and full reference for the retraction notice.*

All the references have been checked and the list has been modified to meet the expected format.

No retracted article has been cited.

**Response to reviewers**

*6. Review Comments to the Author

Please use the space provided to explain your answers to the questions above. You may also include additional comments for the author, including concerns about dual publication, research ethics, or publication ethics. (Please upload your review as an attachment if it exceeds 20,000 characters)*

*Reviewer #1: The authors have addressed my comments and concerns.
The authors have addressed my comments and concerns.*

*Reviewer #2: The authors are to be commended for the thorough work on the revision. This reviewer has the following suggestions for further improvement:

1. General: The language is somewhat suboptimal in certain sections. A proofread by a native English speaker could solve this quite easily.*

The manuscript has been proofread by a native English speaker.

*2. Abstract: "The predefined threshold for scalability (≥0.40) was not achieved for both scales." This should be accompanied by information on the relative lack of variability in the data.*

The mention of the lack of variability in the data has been added in the abstract to explain that scalability was not demonstrated for the scales: **“*Owing to low score variability between patients, the predefined threshold for scalability (≥0.40) was not reached for both scales.*”**.

*3. Methods: A Mokken analysis of the two PDAS subscales (HAM-D6 and BPRS5) would be of interest given the results of the primary analysis (rather low coefficient of homogeneity).*

The Loevinger coefficients of homogeneity derived from the Mokken analyses were 0.39 for HAM-D6 and 0.33 for BPRS5. They were lower than those observed in the original PDAS validation study (0.51 and 0.40 respectively)^[1]^.

*4. Methods: How were PDAS ratings "formatted" for the Mokken analysis (rescaled or)?*

The instructions given in the original article were followed: “*The BPRS item scores (from 1 to 7) were converted to a score between 0 and 4 according to this formula: (BPRS score – 1) x 2/3. For the Mokken analysis, which requires data on a numeric scale, the BPRS scores were converted as follows: 1 = 0, 2–3 = 1, 4-5 = 2, 6 = 3, and 7 = 4. In all analyses of the seven composite scales, the rating on Hamilton item 13 (general somatic) was multiplied by 2*”.

We agree that the method section of our article was not clear enough and we therefore differentiated the method of calculating the total score in the presentation of the PDAS section and that used for the Mokken analysis in the statistical analyses section:

*The PDAS instrument*

***“The total PDAS score is obtained by adding up the sum of the individual item scores after converting the BPRS item scores to a score between 0 and 4 with the formula (BPRS score – 1) x 2/3, and multiplying the score for item 13 on the Hamilton scale (general somatic) by 2.”***

*Statistical analyses*

***“(i)	Scalability was assessed by Mokken analysis, generating a Loevinger coefficient of homogeneity for the PDAS total score. In the Mokken analysis, a Loevinger coefficient of homogeneity ≥0.40 is indicative of scalability [32]. For the present analysis the BPRS scores were converted as follows: 1 = 0, 2–3 = 1, 4-5 = 2, 6 = 3, and 7 = 4.”***

*5. Discussion: "Surprisingly, despite a highly significant result, the Mokken analysis did not confirm the scalability of the PDAS (Loevinger coefficient < 0.40). During the validation of the semi-structured PDAS interview, Köse Çinar and Østergaard [42] also failed to demonstrate the scalability of the PDAS (Loevinger coefficient 0.17) at baseline. They mentioned that it may be due to low variability in PDAS scores (22.8 ± 5.7)."
Yes - but at endpoint in the Köse Çinar and Østergaard study - the Loevinger coefficient was 0.45. This needs to be underlined (fits the explanation). I also think that it deserves mentioning that all four studies having previously assessed the scalability of the PDAS, have confirmed the scalability of the instrument:*
[*https://pubmed.ncbi.nlm.nih.gov/23799875/*](https://pubmed.ncbi.nlm.nih.gov/23799875/)
[*https://pubmed.ncbi.nlm.nih.gov/29501075/*](https://pubmed.ncbi.nlm.nih.gov/29501075/)
[*https://pubmed.ncbi.nlm.nih.gov/25462426/*](https://pubmed.ncbi.nlm.nih.gov/25462426/)
[*https://pubmed.ncbi.nlm.nih.gov/28535843/*](https://pubmed.ncbi.nlm.nih.gov/28535843/) *This leads this reviewer to think, that the present sample of patients is "qualitatively different" from prior studies (see also comment 6B below).*

This section has been completed as requested: “***In general, studies assessing the scalability of the PDAS demonstrate its scalability [16,42,47] and even Köse Çinar and Østergaard found a Loevinger coefficient > 0.40 at endpoint of their study [41].***”

*6. Discussion:* ***"Our data showed similar score with low variability (21.8 ± 4.9), compared to the original PDAS validation which showed higher score and variability (29.5 ± 11.3) [48]. These differences in PDAS score and variability may be due to the population. Despite the same inclusion criteria than the article of Østergaard [17], our population had lower HDRS score, more hallucinations, more suicide attempts and are all inpatients."*** Voir texte modifié *A: What is meant by "similar score" here?*

The sentence has been changed to be clearer: **“*Our data showed a score with low variability (21.8 ± 4.9) close to that in their study [41] whereas the original PDAS validation study showed a higher score and variability (29.5 ± 11.3) [47].*”**

*B: "These differences in PDAS score and variability may be due to the population. Despite the same inclusion criteria than the article of Østergaard [17], our population had lower HDRS score, more hallucinations, more suicide attempts"
This description leads this reviewer to think that there may be a fraction of the participants suffering from either a primary psychotic disorder (ICD-10: F20) or PTSD (as also suggested by one of the other reviewers).*

Psychotic features in depressive disorder should be distinguished from delusional ideas and hallucinations that occur in schizophrenia, and flashbacks in PTSD should be distinguished from hallucinations that can occur in depressive disorder with psychotic features. Several other symptoms support the differential diagnosis between depressive disorder with psychotic features and schizophrenia or PTSD. As our patients were hospitalized in psychiatric units, they were assessed by hospital psychiatrists independent of the study who made a diagnosis during care. For recruitment, we specified that the patients who could be invited to participate in the study were those with a diagnosis of major depressive disorder with psychotic features. In addition, the study psychiatrist systematically checked the inclusion and exclusion criteria.

In our study, the hallucinations and the lower educational level of patients are correlated (please see attached table). This is consistent with the literature (Jääskeläinen E et al, 2018^[2]^). In our study, 70 of the 94 patients were recruited from the psychiatric hospital centres of Clermont-Ferrand and Le Puy en Velay. These hospital centres treat patients with a lower educational level (only 17% of these patients spent more than 12 years in school) who often live in rural communities. The educational level of our patients was lower than the average educational level of patients in the studies of Meyers et al (2006) and SC Park et al (2014). This point has been emphasized in the conclusion: **“*However, other studies are needed to confirm the scalability of the PDAS and the generalisability of the results to a larger population.*”**

Table: SAPS hallucination score according to educational level

| Educational level | SAPS hallucination score (mean ± SD) |
| --- | --- |
| < 12 years | 2.3 ± 1.8 |
| 12 years | 1.7 ± 1.9 |
| > 12 years | 1.1 ± 1.8 |

1. Østergaard SD, Pedersen CH, Uggerby P, Munk-Jørgensen P, Rothschild AJ, Larsen JI, et al. Clinical and psychometric validation of the psychotic depression assessment scale. J Affect Disord. 1 mars 2015;173:261‑8.
2. Jääskeläinen E, Juola T, Korpela H, Lehtiniemi H, Nietola M, Korkeila J, et al. Epidemiology of psychotic depression - systematic review and meta-analysis. Psychol Med. 2018;48(6):905‑18.
